# Cluster‐Defined Response Patterns Following Pain Neuroscience Education With Physical Activity in Older Women With Chronic Low Back Pain: A Quasi‐Randomized Exploratory Study

**DOI:** 10.1002/hsr2.73241

**Published:** 2026-09-25

**Authors:** Teppei Abiko, Shin Murata, Hiroaki Iwase, Koji Nonaka, Kunihiko Anami, Yuki Kikuchi, Katsuyuki Madoba, Kayoko Shiraiwa, Guillermo de Castro Maqueda, Wayne Hing

**Affiliations:** ^1^ Faculty of Health Sciences and Medicine Bond University Gold Coast Queensland Australia; ^2^ Department of Physical Therapy, Faculty of Health Sciences Kyoto Tachibana University Kyoto Kyoto Japan; ^3^ Department of Physical Therapy, Faculty of Rehabilitation Science Kobe International University Kobe Hyogo Japan; ^4^ Department of Rehabilitation, Faculty of Health Sciences Naragakuen University Nara Nara Japan; ^5^ Department of Rehabilitation Nishikyoto Hospital Kyoto Kyoto Japan; ^6^ Department of Physical Education, School of Education Science University of Cádiz Cádiz Cádiz Spain

**Keywords:** chronic low back pain, older women, pain neuroscience education

## Abstract

**Background and Aims:**

To explore patterns of treatment response to pain neuroscience education combined with physical activity (PNE‐PA) and to examine baseline characteristics associated with these patterns in older women with chronic low back pain (CLBP).

**Methods:**

In a community‐based quasi‐randomized trial, older women with CLBP were analyzed in a PNE‐PA program (*n* = 43) or a biomechanical (BM) comparison group (*n* = 13). Physical function, physical activity (daily step counts by pedometer), pain intensity, disability, and pain‐related psychological outcomes were assessed at baseline and 12 weeks. Between‐group effects were examined using rank‐based analysis of covariance adjusting for baseline values, age, and body mass index. Within the PNE‐PA arm, k‐means clustering on changes in 30‐s chair‐stand performance and pain catastrophizing (PCS) was used to identify response profiles, with baseline characteristics compared across clusters.

**Results:**

Compared with BM, PNE‐PA produced larger adjusted improvements in chair‐stand repetitions, daily step counts, and PCS scores; no significant between‐group differences were observed for other outcomes. Three clusters were identified: minimal responders (*n* = 15), physical‐function responders (*n* = 18), and psychological responders (*n* = 10). Physical‐function responders showed greater chair‐stand gains with lower baseline lower‐limb function; psychological responders exhibited larger PCS reductions and lower baseline physical performance, together with higher PCS scores. Cluster separation was modest, and the gap statistic favored a single cluster, indicating descriptive, hypothesis‐generating patterns.

**Conclusion:**

Older women with CLBP receiving PNE‐PA showed heterogeneous changes in physical function and pain‐related cognitions. Lower baseline mobility and higher catastrophizing were associated with larger domain‐specific improvements, although these associations may partly reflect regression to the mean. Identifying baseline physical and psychological profiles may inform more targeted group‐based programs for older adults with CLBP.

## 1 Introduction

Chronic low back pain (CLBP) is one of the most prevalent musculoskeletal disorders among community‐dwelling older adults, contributing to significant reductions in health‐related quality of life [1]. As populations age, the burden of CLBP is expected to grow, particularly among older women who show a higher prevalence and greater disability than men of the same age. In this often‐independent population, persistent pain frequently interferes with daily functioning, reduces physical activity, and exacerbates psychological distress [2, 3]. The persistence of CLBP involves not only physical but also psychosocial factors. According to the fear‐avoidance model proposed by Vlaeyen et al. [4], psychological responses such as pain catastrophizing and fear of movement lead to activity avoidance, further limiting physical activity. In older adults, such inactivity may accelerate frailty progression and contribute to social withdrawal or homebound status. Several studies demonstrated that fear related to pain was significantly associated with decreased physical activity levels in older individuals with CLBP [5, 6, 7]. Physical activity has also been shown to directly reduce pain intensity and improve disability in older adults with CLBP [8]. Therefore, early intervention targeting both physical and psychological factors is crucial not only for pain management but also for preventing frailty and social isolation in aging populations.

Pain neuroscience education (PNE) has been introduced as an educational intervention aimed at modifying maladaptive beliefs and promoting adaptive pain coping strategies [9]. By explaining the neurophysiological mechanisms of pain, PNE seeks to reduce pain‐related fear and pain catastrophizing. Its effectiveness in reducing pain intensity, functional disability, and psychological distress has been well documented in younger and middle‐aged adults [10, 11]. However, evidence for its efficacy in older adults remains limited [12, 13, 14]. Specifically, the validation of PNE in older adults is limited, with insufficient investigation into the variability of its effects or which older individuals would benefit most. Moreover, existing studies indicate that not all patients benefit equally from PNE, highlighting the importance of understanding individual variability in treatment response [15, 16]. Identifying responders and characterizing their baseline features may provide valuable information for informing the design of more efficient group‐based interventions, particularly in community settings where individualized approaches are often impractical or resource‐intensive.

Given these aforementioned considerations, the primary aim of this study was not only to evaluate the effects of PNE combined with physical activity in community‐dwelling older adults with CLBP but also to explore distinct response patterns and identify baseline characteristics associated with favorable treatment responses. Such knowledge can inform the development of more efficient, targeted, and resource‐sensitive interventions for older adults experiencing chronic pain.

## 2 Materials and Methods

### 2.1 Study Design

This quasi‐randomized controlled trial examined the effects of PNE combined with physical activity (PNE‐PA) and explored individual response patterns in community‐dwelling older women with CLBP. Participants were allocated to the intervention arms according to the recruitment period because of logistical constraints on group‐based delivery [17]. Specifically, participants recruited during period 1, October 2017–December 2017, were assigned to the BM group, those recruited during period 2, October 2018–December 2018, and period 3, October 2019–December 2019, were assigned to the PNE‐PA group; the recruitment periods of the two groups did not overlap. Although this allocation method introduces the possibility of time‐ and season‐related confounding, both groups completed assessments within the same calendar year, and no major changes in program delivery occurred across periods. Outcome assessors were blinded to group assignment, and participants were not informed about between‐group comparisons. The study adhered to the Declaration of Helsinki and was approved by the Research Ethics Committee of Kyoto Tachibana University (Approval No. 15‐11). All participants provided written informed consent. It was registered with the University Hospital Medical Information Network (UMIN000044436).

### 2.2 Subjects

Eligible participants were community‐dwelling women aged 65 years or older who reported CLBP for more than 3 months. CLBP was defined as low back pain persisting for at least 3 months, without subclassification by structural diagnosis. This study focused on women because epidemiological studies have consistently shown a higher prevalence and disability burden of CLBP in older women than in men, and because sex‐specific physiological and psychosocial factors may influence pain and aging trajectories [18].

Participants were excluded if they presented with neurological symptoms (such as radiculopathy or spinal stenosis), major systemic illnesses (including cardiac, respiratory, rheumatologic, immunologic, or oncologic disease), or cognitive impairment (Mini‐Mental State Examination score < 24). Furthermore, participants were excluded if they were routinely using pain medication, had other musculoskeletal conditions judged likely to influence intervention effects, or had missing data for any primary outcome at either baseline or post‐intervention. Regular analgesic use was specifically excluded to reduce pharmacological heterogeneity and ensure the improved interpretability of the behavioral and educational effects on pain‐related outcomes. Data collection took place at the Yamashina Central Elderly Welfare Center in Kyoto, Japan.

### 2.3 Interventions

Participants were then assigned to one of two interventions: PNE‐PA or a biomechanical approach (BM) (Table 1). The BM group was implemented as an active control to specifically separate the psychological components of PNE‐PA from non‐psychological mechanisms, such as biomechanical influences, involved in CLBP treatment. Both programs were conducted face‐to‐face in group settings, consisting of six sessions, each lasting 60 min, held every 2 weeks over a 12‐week period. All participants were instructed to perform daily home exercises and document compliance using a logbook.

**Table 1:** Description of both interventions.

PNE‐PA	BM
(Pain neuroscience education plus physical activity)	(Biomechanical approach)
**Intervention Duration and Format**	
Six face‐to‐face group sessions over 12 weeks, held every 2 weeks.	
Each session was 60 min long.	
**Facilitator**	
A licensed physical therapist with over a decade of clinical experience and prior training in PNE.	
**Session Content**	
The 60‐min session was evenly split between interactive PNE and practical guidance on facilitating physical activity.	Consisted of a 30‐min educational session followed by a 30‐min exercise component.
**Education Component**	
Emphasized neurophysiological principles of pain, including the distinction between acute and chronic pain, descending inhibitory pathways, central sensitization, and neuroplasticity.	Covered precautions during daily movements, biomechanical explanations for LBP, LBP subtypes, and corresponding management.
Highlighted the influence of psychological and environmental factors on pain.	Taught principles of conventional lumbar care, including maintaining a neutral lumbar spine posture and minimizing excessive flexion and extension.
Used simplified diagrams, brochures, and metaphors (e.g., the alarm system) to explain complex concepts.	Provided practical strategies for spine protection and postural control during daily tasks.
Employed repetitive teaching strategies like frequent questioning and quizzes to aid comprehension and memory, specifically for older participants.	
Discouraged excessive focus on posture, instead contextualizing pain within common geriatric concerns (e.g., chronic inflammation, osteoporosis).	
**Physical activity/exercise component**	
Reframed the role of physical activity to include nervous system regulation, emotional well‐being, and general health, not just musculoskeletal strengthening.	Individualized based on the participant's LBP presentation (flexion‐pattern or extension‐pattern).
Participants used a daily logbook to track step counts and personally meaningful activities to monitor progress and maintain motivation.	For flexion‐pattern LBP: Incorporated strengthening exercises for the iliopsoas and paraspinal muscles, along with global body extension exercises.
Walking was promoted using step count as a metric, with no fixed targets; participants were encouraged to increase activity at their own pace.	For extension‐pattern LBP: Included stretching protocols for the iliopsoas and paraspinal musculature, and core stabilization exercises focused on strengthening abdominal muscles.
	Participants were instructed to perform strengthening exercises in 3 sets of 10–15 repetitions and hold stretches for 30 sec, three times daily at home.
	Illustrated handouts for each exercise were distributed.

Abbreviations: BM, biomechanical approach; LBP, low back pain; PNE‐PA, pain neuroscience education plus physical activity.

The PNE‐PA program consisted of six 60‐min group sessions delivered every 2 weeks over 12 weeks. Each session devoted approximately 30 min to interactive PNE and 30 min to promoting physical activity. Educational content addressed the neurophysiology of pain, including differences between acute and chronic pain, central sensitization, and the influence of psychological and environmental factors. Complex concepts were conveyed using simplified diagrams, analogies, and visual materials, and key messages were reinforced through repetition, questioning, and short quizzes to suit older participants. Posture was de‐emphasized to avoid reinforcing structural fear, and pain was contextualized within common age‐related conditions such as chronic inflammation and osteoporosis. Physical activity was framed as a means not only of strengthening but also of modulating nervous system sensitivity, mood, and general health. Participants recorded daily step counts and personally meaningful activities in logbooks and were encouraged to gradually increase activity at a pace they perceived as safe. Sessions were led by a licensed physical therapist with extensive experience in PNE and geriatric practice.

The BM program also comprised six 60‐min group sessions. The first 30 min focused on biomechanical education, including spinal anatomy, classification of LBP presentations, and conventional advice regarding neutral spinal alignment and avoidance of extremes of flexion and extension. The subsequent 30 min consisted of exercises individually tailored to flexion‐ or extension‐dominant LBP patterns. Flexion‐related pain was addressed through strengthening exercises for the iliopsoas and paraspinal muscles plus whole‐body extension tasks, whereas extension‐related pain was addressed with stretching of these muscles and strengthening of abdominal musculature. Participants were instructed to perform three sets of 10–15 repetitions for strengthening exercises and to hold stretches for 30 s, three times daily. Exercise sheets and logbooks were provided. BM sessions were delivered by a physical therapist with a doctoral degree in musculoskeletal physiotherapy.

## 3 Measurements

### 3.1 Outcome Assessment

All participants underwent a series of physical performance assessments and completed demographic forms along with standardized self‐report questionnaires related to pain and psychological factors (Table 2). These evaluations were conducted at two time points: prior to the intervention (baseline) and immediately after the 12‐week intervention period.

**Table 2:** Classification of outcome measures by functional domain.

Category	Assessment tool	Purpose
Physical Function	Grip Strength	Muscle Strength
	Sit‐and‐Reach Test	Flexibility
	One‐Leg Stand Test	Static Balance
	30‐Second Chair‐Stand Test	Lower Limb Strength/Balance
	Timed Up and Go Test	Dynamic Balance
Physical Activity	Step Counts	Activity Level
Pain‐Related Questionnaire	Visual Analog Scale	Pain Intensity
	Roland‐Morris Disability Questionnaire	Pain‐Related Functional Disability
Psychological Factors	Pain Catastrophizing Scale	Pain Catastrophizing
	Fear Avoidance Beliefs Questionnaire	Fear‐Avoidance Beliefs
	Five‐item Geriatric Depression Scale	Depression

### 3.2 Physical Function

Grip strength, an indicator of general muscular strength and frailty risk, was measured using a calibrated handheld dynamometer. Each hand was tested twice, and the maximum value for each was recorded. Flexibility was assessed with the Sit‐and‐Reach Test (SRT), a standard measure of hamstring and lower back flexibility. Participants sat with legs extended and reached forward along a measuring line as far as possible without bending their knees [19]. The furthest distance reached was used for analysis. Balance ability was measured by the One‐Leg Stand Test (OLS). Participants stood on one leg with eyes open and arms by their sides, and the time they were able to maintain the position without support was recorded, up to a maximum of 120 s [20]. Lower‐limb muscle strength and balance ability were assessed using the 30‐Second Chair‐Stand Test (CS30), in which participants were instructed to rise to a full standing position and return to a seated position as many times as possible within 30 s [21]. The number of completed repetitions was recorded as an index of lower‐extremity function. Dynamic balance was evaluated using the Timed Up and Go (TUG). Participants were timed as they rose from a chair, walked 3 m, turned around, returned to the chair, and sat down [22]. The total time taken reflected functional mobility and dynamic balance.

### 3.3 Physical Activity

Physical activity levels were monitored using a pedometer (YAMASA EX‐500, Japan), which recorded daily step counts. Participants were instructed to wear the pedometer on the waist throughout the day and record their step count in a daily logbook. For analysis, the average daily step count was calculated from entries during the first and twelfth weeks of the intervention period.

### 3.4 Pain and Disability

Pain intensity was assessed using the Visual Analog Scale (VAS) [23], in which participants marked their current level of pain on a 10‐cm horizontal line ranging from “no pain” to “worst pain imaginable.” The distance from the left end to the marked point provided a continuous measure of pain intensity. Functional disability due to LBP was evaluated using the Roland–Morris Disability Questionnaire (RDQ), which consists of 24 dichotomous items reflecting limitations in daily activities [24, 25]. The total score, obtained by summing affirmative responses, indicated the level of disability; higher scores represented greater functional impairment.

### 3.5 Psychological Factors

Pain‐related cognitive and emotional responses were assessed using the Pain Catastrophizing Scale (PCS), a 13‐item measure capturing the extent of rumination, magnification, and helplessness related to pain [26, 27]. Each item was rated on a 5‐point Likert scale from 0 (“not at all”) to 4 (“all the time”), with total scores reflecting the degree of pain catastrophizing. Fear‐avoidance beliefs were measured by the Fear‐Avoidance Beliefs Questionnaire (FABQ), which comprises items addressing beliefs about how physical activity and work affect pain [28, 29]. Respondents rated their agreement on a 7‐point scale ranging from 0 (“completely disagree”) to 6 (“completely agree”), yielding subscale scores that reflect avoidance behavior toward activity or work due to pain. Depressive symptoms were screened using the Five‐item Geriatric Depression Scale (GDS‐5), a concise tool developed for use in older populations [30]. Participants answered five yes/no questions concerning their mood and general outlook over the past week. Higher scores indicated a greater likelihood of depressive symptoms.

### 3.6 Sample Size

This study was designed to explore individual response patterns within the intervention arm rather than to detect prespecified between‐group effects with formal statistical power. Accordingly, no a priori sample size calculation was performed. Because the primary analysis used unsupervised k‐means clustering on changes in CS30 and PCS, we judged sample adequacy with reference to small‐sample guidance for exploratory clustering rather than hypothesis‐testing criteria. In line with these recommendations, we limited the analysis to plausible low‐k solutions (2–3 clusters) and prespecified *k* = 3 for clinical interpretability; clustering results were therefore treated as descriptive and hypothesis‐generating [31]. The intervention group comprised 43 participants, which we deemed sufficient for exploratory identification of response profiles under these constraints. The BM comparison group comprised 13 participants. Given the relatively small control group, between‐group comparisons were likewise considered exploratory.

### 3.7 Statistical Analysis

Given the quasi‐randomized design, modest sample size, and multiple outcomes, all analyses were treated as exploratory and *p*‐values are reported without adjustment for multiplicity; they should therefore be interpreted descriptively rather than as formal hypothesis tests. Item‐level missing data were imputed using multiple imputation by chained equations (MICE 3.17.0; predictive mean matching, *m* = 5, seed = 123). Participants with time‐point missingness of a primary outcome were not included in that outcome's analysis, and imputation was applied only at the item level.

In the first stage, baseline characteristics of the PNE‐PA and BM groups were compared using two‐sided Mann–Whitney *U* tests. For each outcome, we calculated a change score (Δ = post − baseline) and applied Quade's rank ANCOVA to compare PNE‐PA and BM while adjusting for baseline value, age, and body mass index. Adjustment for baseline values was considered essential given that quasi‐randomized allocation by recruitment period was anticipated to yield pre‐existing group differences; this approach ensures that the reported between‐group effects reflect change independent of baseline. We report Quade's F, two‐sided *p*‐values, and nonparametric effect sizes (*ε* ^2^). For Mann–Whitney *U* tests, effect sizes are reported as *r* (0.1/0.3/0.5 ≈ small/medium/large) [32]. For Quade's rank ANCOVA, *ε* ^2^ thresholds of 0.01, 0.06, and 0.14 denote small, moderate, and large effects, respectively. Between‐group analyses were undertaken to describe potential differential change rather than to establish efficacy.

In the second stage (PNE‐PA group), we explored response profiles using cluster analysis. We prespecified *k* = 3 for clinical interpretability and performed *k*‐means on standardized ΔCS30 and ΔPCS. These two variables were selected as clustering dimensions because they represent the primary physical and psychological outcomes targeted by PNE‐PA, respectively, and because their combination captures the dual focus of the intervention on functional improvement and pain‐related cognition. Cluster differences in baseline variables and in changes of secondary outcomes were tested with Kruskal–Wallis tests followed, when significant, by Dunn's tests with Holm adjustment. Cluster stability was summarized by the silhouette coefficient (overall and per cluster). As a sensitivity check, we also computed the gap statistic for *k* = 1–6 and inspected the optimal *k* according to Tibshirani's rule [33]. Because of the limited sample size and modest stability indices, cluster findings were interpreted as descriptive and hypothesis‐generating rather than as definitive subgroup identification.

An a priori significance level of *α* = 0.05 was applied to all tests. All tests were two‐sided unless otherwise stated. Analyses were performed in R (version 4.4.3) with the mice package (version 3.17.0) for multiple imputation.

## 4 Results

### 4.1 Baseline

A total of 75 participants were enrolled (57 in the PNE‐PA group and 18 in the BM group). Of these, 19 were excluded owing to missing post‐intervention outcome data, leaving 56 participants (43 PNE‐PA; 13 BM) in the final analysis. At baseline, the PNE‐PA group was older and showed lower physical function and lower GDS‐5 scores than the BM group (Table 3). Median age was 76 years (IQR 74–80) versus 72 (67–77) (*p* = 0.034, *r* = 0.392). One‐leg stand time was shorter in PNE‐PA (23.1 s [12.1–52.5]) than in BM (88.5 s [61.2–120.0]) (*p* = 0.001, *r* = −0.592), and CS30 repetitions were fewer (20 [15–23] vs. 24 [20–26]) (*p* = 0.028, *r* = −0.406). GDS‐5 scores were lower in PNE‐PA (1 [0–1]) than in BM (2 [1–2]) (*p* = 0.003, *r* = −0.512). Other measures, including BMI, TUG, grip strength, daily steps, pain intensity, RDQ, PCS, and FABQ, did not significantly differ. No adverse events were observed in any of the participants.

**Table 3:** Baseline characteristics of participants in the BM and PNE‐PA groups.

	Group	Median (IQR)	*p* value	Effect size (*r*)
Age	BM	72.00 [67.00–77.00]	0.034	0.392
	PNE‐PA	76.00 [74.00–80.00]		
Height	BM	150.70 [147.50–151.90]	0.560	0.109
(cm)	PNE‐PA	150.30 [148.00–154.00]		
Weight	BM	46.60 [42.50–50.70]	0.056	0.354
(kg)	PNE‐PA	50.70 [45.35–55.15]		
BMI	BM	20.18 [19.12–22.41]	0.084	0.320
	PNE‐PA	22.12 [20.12–23.47]		
Grip Strength	BM	23.10 [22.90–25.40]	0.264	−0.208
(kg)	PNE‐PA	22.50 [19.35–24.65]		
SRT	BM	34.00 [30.50–39.00]	0.145	−0.270
(cm)	PNE‐PA	30.50 [23.50–38.75]		
OLS	BM	88.45 [61.24–120.00]	0.001	−0.592
(sec)	PNE‐PA	23.06 [12.05–52.53]		
CS30	BM	24.00 [20.00–26.00]	0.028	−0.406
(reps)	PNE‐PA	20.00 [15.00–23.00]		
TUG	BM	5.50 [5.20–6.50]	0.076	0.329
(sec)	PNE‐PA	6.34 [5.59–7.09]		
Step Counts	BM	4392.00 [3422.00–5461.00]	0.103	0.302
(steps/day)	PNE‐PA	5544.29 [4323.50–6396.50]		
Pain Intensity	BM	20.00 [20.00–40.00]	0.690	0.075
(points)	PNE‐PA	27.00 [18.50–49.50]		
RDQ	BM	2.00 [1.00–5.00]	0.569	0.106
(points)	PNE‐PA	2.00 [1.00–6.50]		
PCS	BM	20.00 [13.00–33.00]	0.915	0.021
(points)	PNE‐PA	21.00 [15.00–31.00]		
FABQ	BM	12.00 [7.00–18.00]	0.087	0.317
(points)	PNE‐PA	16.00 [14.00–20.00]		
GDS‐5	BM	2.00 [1.00–2.00]	0.003	−0.512
(points)	PNE‐PA	1.00 [0.00–1.00]		

*Note:* Data are presented as median [IQR]. Between‐group comparisons used Mann–Whitney *U*.

Effect size (*r*) interpretation: small = 0.1, medium = 0.3, large = 0.5.

Abbreviations: BM, biomechanical approach; BMI, body mass index; CS30, 30‐Second Chair‐Stand Test; FABQ, Fear‐Avoidance Beliefs Questionnaire; GDS‐5, Five‐item Geriatric Depression Scale; IQR, interquartile range; OLS, One‐Leg Stand Test; PCS, Pain Catastrophizing Scale; PNE‐PA, Pain Neuroscience Education plus Physical Activity; RDQ, Roland–Morris Disability Questionnaire; SRT, Sit‐and‐Reach Test; TUG, Timed Up and Go test.

### 4.2 Intervention Effects

After adjustment for baseline value, age, and BMI using Quade's rank ANCOVA, PNE‐PA showed greater improvements in lower‐limb function, daily steps, and PCS than BM (Table 4). CS30 increased by 2.0 repetitions (IQR 0.0–5.0) in PNE‐PA but decreased by 4.0 (−5.2–0.0) in BM (*F* = 12.10, *p* = 0.001, *ε* ^2^ = 0.168). Daily steps rose by 68.9 (−462.0–955.0) under PNE‐PA and declined by 408.0 (−708.0 to −15.0) under BM (*F* = 4.82, *p* = 0.033, *ε* ^2^ = 0.065). PCS decreased by 6.0 points (−12.5–0.0) in PNE‐PA but increased by 3.0 (−1.0–6.0) in BM (*F* = 5.93, *p* = 0.018, *ε* ^2^ = 0.082). No significant between‐group differences were observed in pain intensity (VAS), disability (RDQ), grip strength, flexibility (SRT), static balance (OLS), dynamic balance (TUG), fear‐avoidance beliefs (FABQ), or depressive symptoms (GDS‐5) (Table 4).

**Table 4:** Adjusted between‐group differences in outcomes following intervention.

Outcome	Group	Raw median (IQR)	*F*‐value	*p*‐value	Effect size (*ε* ^2^)
ΔGrip strength	BM	−0.40 [−0.80–0.60]	0.379	0.540	< 0.001
	PNE‐PA	0.30 [−1.10–1.85]			
ΔSRT	BM	−2.00 [−4.50–2.50]	1.311	0.257	0.006
	PNE‐PA	1.50 [−4.00–4.50]			
ΔOLS	BM	0.00 [−13.48–24.00]	0.894	0.349	< 0.001
	PNE‐PA	2.06 [−2.84–22.57]			
ΔCS30	BM	−4.00 [−5.15–0.00]	12.098	0.001	0.168
	PNE‐PA	2.00 [0.00–5.00]			
ΔTUG	BM	0.10 [−0.20–0.20]	0.235	0.630	< 0.001
	PNE‐PA	0.08 [−0.94–0.44]			
ΔStep Counts	BM	−408.00 [−708.00–−15.00]	4.816	0.033	0.065
	PNE‐PA	68.91 [−462.00–955.00]			
ΔPain Intensity	BM	−10.00 [−20.00–0.00]	1.489	0.228	0.009
	PNE‐PA	−4.00 [−16.00–6.00]			
ΔRDQ	BM	0.00 [−2.00–0.00]	2.039	0.159	0.019
	PNE‐PA	−1.00 [−2.00–0.00]			
ΔPCS	BM	3.00 [−1.00–6.00]	5.926	0.018	0.082
	PNE‐PA	−6.00 [−12.50–0.00]			
ΔFABQ	BM	1.00 [−3.00–5.00]	0.186	0.668	< 0.001
	PNE‐PA	−3.00 [−9.50–3.50]			
ΔGDS‐5	BM	0.00 [−1.00–0.00]	0.101	0.752	< 0.001
	PNE‐PA	0.00 [0.00–0.00]			

*Note:* Median changes and between‐group comparisons based on Quade's rank ANCOVA.

Effect size (*ε* ^2^) interpretation: small = 0.01, moderate = 0.06, large = 0.14.

Abbreviations: Δ, change score (post‐intervention minus baseline); ΔGrip strength, change in grip strength; ΔSRT, change in Sit‐and‐Reach Test; ΔOLS, change in One‐Leg Stand Test; ΔCS30, change in 30‐Second Chair‐Stand Test; ΔTUG, change in Timed Up and Go test; ΔStep Counts, change in mean daily steps; ΔPain Intensity, change in Visual Analog Scale; ΔRDQ, change in Roland–Morris Disability Questionnaire; ΔPCS, change in Pain Catastrophizing Scale; ΔFABQ, change in Fear‐Avoidance Beliefs Questionnaire; ΔGDS‐5, change in Five‐item Geriatric Depression Scale.

### 4.3 Response Profiles (PNE‐PA Only)

**Figure 1:**
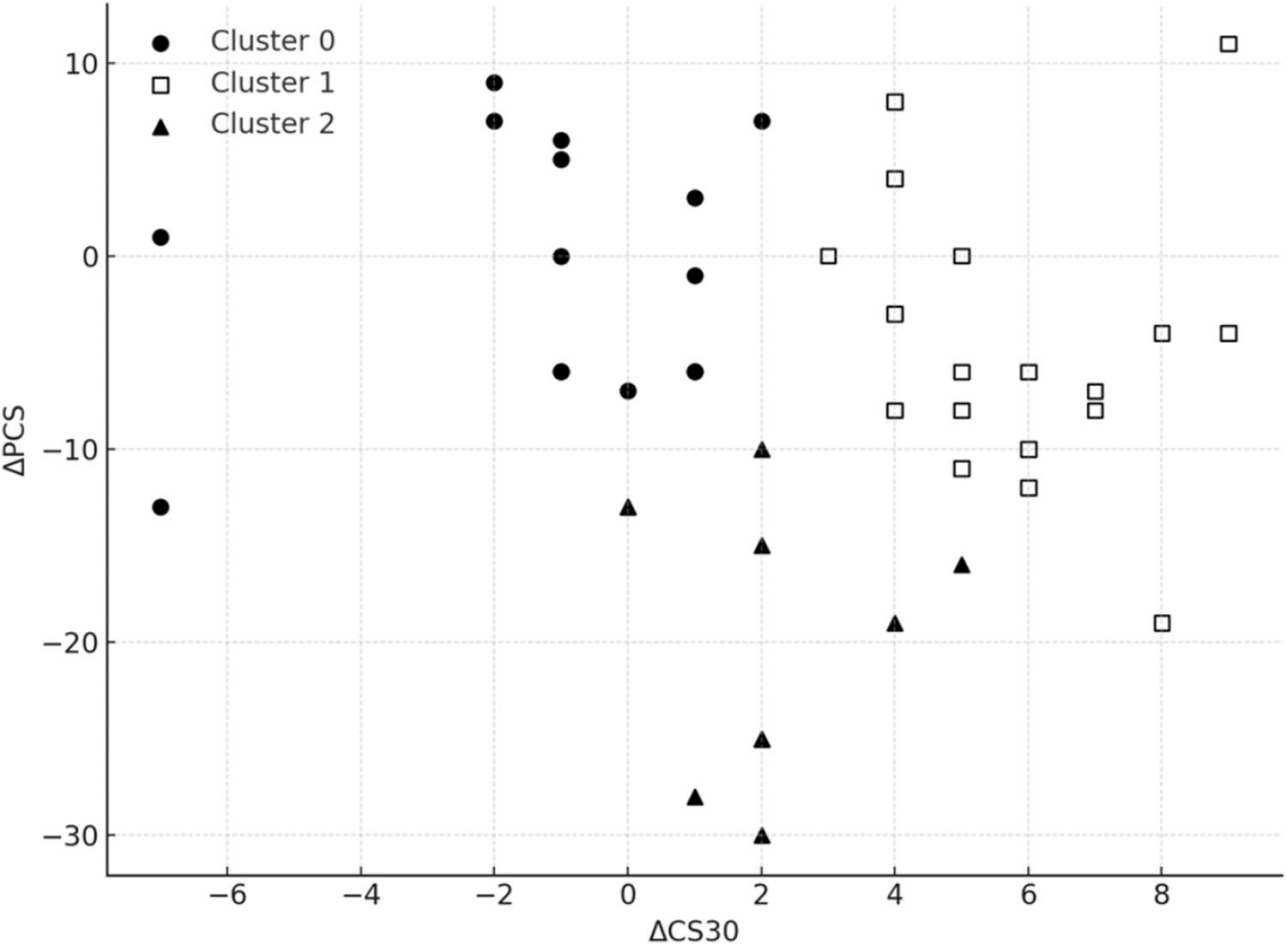
Cluster Classification of PNE‐PA Responders Based on Changes in Chair‐Stand Performance (ΔCS30) and Pain Catastrophizing (ΔPCS). The *x*‐axis represents ΔCS30 (change in 30‐Second Chair‐Stand Test repetitions; post‐intervention minus baseline) and the *y*‐axis represents ΔPCS (change in Pain Catastrophizing Scale score; post‐intervention minus baseline). Each symbol represents one participant, with symbol shape/color denoting cluster membership. Cluster 0: Minimal Responders (*n* = 15); Cluster 1: Physical Function Responders (*n* = 18); Cluster 2: Psychological Responders (*n* = 10). Positive ΔCS30 values indicate improvement in lower‐limb function; negative ΔPCS values indicate reduction in pain catastrophizing.

K‐means clustering of ΔCS30 and ΔPCS identified three subgroups (Cluster 0 [C0], *n* = 15; Cluster 1 [C1], *n* = 18; Cluster 2 [C2], *n* = 10) (Figure 1): Minimal Responders (Cluster 0), Physical Function Responders (Cluster 1), and Psychological Responders (Cluster 2). ΔCS30 differed across clusters (*H* = 32.73, *p* < 0.001), with Physical Function Responders outperforming Minimal and Psychological Responders. ΔPCS also differed (*H* = 21.88, *p* < 0.001): Psychological Responders had the largest PCS reduction (−17.5 [−25.0 to −13.5]), Physical Function Responders showed a moderate reduction (−6.0 [−8.0 to −0.8]), and Minimal Responders showed little change (0.0 [−6.0 to 5.5]) (Table 5). The PCS reduction in Psychological Responders exceeded that in the other two clusters.

**Table 5:** Changes in outcome measures across identified response clusters in the PNE‐PA group.

Variable	Cluster	Median (IQR)	*H*	*p*‐value	Multiple comparison
ΔGrip strength	0	−0.80 [−2.10–0.95]	4.874	0.087	
	1	0.50 [−0.40–1.93]			
	2	1.25 [−0.42–2.58]			
ΔSRT	0	0.50 [−4.50–3.25]	1.264	0.532	
	1	1.75 [−4.00–6.75]			
	2	3.00 [−2.50–4.25]			
ΔOLS	0	2.06 [−4.56–23.38]	0.095	0.953	
	1	3.48 [−4.30–27.22]			
	2	1.62 [−0.31–12.05]			
ΔCS30	0	−1.00 [−1.50–1.00]	32.729	< 0.001	0, 2 < 1
	1	5.50 [4.25–7.00]			
	2	2.00 [1.25–2.00]			
ΔTUG	0	0.35 [−0.63–0.43]	3.602	0.165	
	1	−0.54 [−1.42–0.27]			
	2	0.28 [−0.15–0.53]			
ΔStep Counts	0	861.71 [−1009.86–1541.43]	0.796	0.672	
	1	189.40 [−398.68–655.61]			
	2	190.25 [189.60–212.03]			
ΔPain intensity	0	2.00 [−8.50–6.00]	1.639	0.441	
	1	−5.00 [−16.00–7.75]			
	2	−8.50 [−14.25–−3.25]			
ΔRDQ	0	0.00 [−1.00–0.00]	2.056	0.358	
	1	−1.50 [−2.00–0.00]			
	2	−1.00 [−2.00–0.00]			
ΔPCS	0	0.00 [−6.00–5.50]	21.884	< 0.001	2 < 0, 1
	1	−6.00 [−8.00–−0.75]			
	2	−17.50 [−25.00–−13.50]			
ΔFABQ	0	−1.00 [−6.00–5.00]	1.519	0.468	
	1	−4.00 [−8.00–−0.25]			
	2	−5.00 [−11.75–2.50]			
ΔGDS‐5	0	0.00 [−1.00–0.00]	2.701	0.259	
	1	0.00 [0.00–0.00]			
	2	0.00 [−0.75–0.00]			

*Note:* Cluster 0: Minimal Responders; Cluster 1: Physical Function Responders; Cluster 2: Psychological Responders.

Abbreviations: Δ, change score (post‐intervention minus baseline); ΔGrip strength, change in grip strength; ΔSRT, change in Sit‐and‐Reach Test; ΔOLS, change in One‐Leg Stand Test; ΔCS30, change in 30‐Second Chair‐Stand Test; ΔTUG, change in Timed Up and Go test; ΔStep Counts, change in mean daily steps; ΔPain Intensity, change in Visual Analog Scale; ΔRDQ, change in Roland–Morris Disability Questionnaire; ΔPCS, change in Pain Catastrophizing Scale; ΔFABQ, change in Fear‐Avoidance Beliefs Questionnaire; ΔGDS‐5, change in Five‐item Geriatric Depression Scale.

### 4.4 Baseline Differences Between Clusters (PNE‐PA Only)

At baseline, clusters differed in SRT (*H* = 7.49, *p* = 0.024), OLS (*H* = 8.61, *p* = 0.014), and CS30 repetitions (*H* = 10.60, *p* = 0.005) (Table 6). Psychological Responders exhibited the lowest physical function across these measures. For CS30 specifically, both Psychological and Physical Function Responders scored lower than Minimal Responders, with Psychological Responders recording the fewest repetitions. Other baseline variables—including age, BMI, grip strength, TUG, step counts, pain intensity, RDQ, FABQ, and GDS‐5—did not differ (all *p* > 0.05). Baseline PCS showed a non‐significant trend (*p* = 0.086), with Psychological Responders having the highest catastrophizing.

**Table 6:** Baseline characteristics of participants stratified by response cluster in the PNE‐PA group.

Variable	Cluster	Median (IQR)	*H*	*p*‐value	Multiple comparison
Age	0	76.00 [72.50–78.00]	4.676	0.096	
	1	74.50 [72.50–77.00]			
	2	79.50 [79.00–81.75]			
BMI	0	20.34 [19.54–21.36]	0.912	0.634	
	1	22.55 [21.28–23.49]			
	2	22.20 [20.54–23.58]			
Grip_baseline	0	22.90 [21.70–24.65]	2.356	0.308	
	1	21.15 [18.70–24.77]			
	2	20.75 [18.68–23.95]			
SRT_baseline	0	32.50 [27.50–40.25]	7.491	0.024	2 < 0
	1	32.25 [26.75–39.38]			
	2	23.00 [17.00–28.25]			
OLS_baseline	0	35.02 [17.86–52.53]	8.607	0.014	2 < 0, 1
	1	30.00 [16.18–88.18]			
	2	8.25 [4.30–19.43]			
CS30_baseline	0	23.00 [20.50–26.00]	10.604	0.005	1, 2 < 0
	1	19.50 [14.50–21.50]			
	2	15.00 [13.25–17.75]			
TUG_baseline	0	6.00 [5.44–6.50]	2.693	0.260	
	1	6.34 [5.65–7.55]			
	2	6.91 [5.88–7.59]			
Steps_baseline	0	5417.43 [3514.64–7183.14]	0.759	0.684	
	1	5787.61 [4987.68–7231.75]			
	2	5786.94 [4781.31–5788.54]			
Pain Intensity baseline	0	27.00 [16.00–44.00]	0.476	0.788	
	1	25.50 [20.00–41.25]			
	2	34.00 [20.25–49.75]			
RDQ_baseline	0	1.00 [0.00–7.00]	2.121	0.346	
	1	3.50 [2.00–5.75]			
	2	2.50 [2.00–7.25]			
PCS_baseline	0	19.00 [6.00–27.00]	4.901	0.086	
	1	20.00 [15.75–33.00]			
	2	30.50 [23.25–32.50]			
FABQ_baseline	0	16.00 [14.50–19.00]	0.251	0.882	
	1	17.00 [15.00–20.00]			
	2	17.00 [12.00–23.25]			
GDS‐5_baseline	0	1.00 [1.00–1.50]	0.757	0.685	
	1	1.00 [0.00–1.75]			
	2	1.00 [0.25–1.00]			

*Note:* Cluster 0: Minimal Responders; Cluster 1: Physical Function Responders; Cluster 2: Psychological Responders.

Abbreviations: BMI, body mass index; CS30_baseline, baseline 30‐Second Chair‐Stand Test; FABQ_baseline, baseline Fear‐Avoidance Beliefs Questionnaire; GDS‐5_baseline, baseline Five‐item Geriatric Depression Scale; Grip_baseline, baseline grip strength; OLS_baseline, baseline One‐Leg Stand Test; PCS_baseline, baseline Pain Catastrophizing Scale; Pain Intensity baseline, baseline Visual Analog Scale; RDQ_baseline, baseline Roland–Morris Disability Questionnaire; SRT_baseline, baseline Sit‐and‐Reach Test; Step_baseline, baseline mean daily step counts; TUG_baseline, baseline Timed Up and Go test.

### 4.5 Cluster Stability

Cluster sizes were C0 = 15, C1 = 18, and C2 = 10 (*N* = 43). Cluster profiles for the primary clustering variables were as follows: median ΔCS30 = −1.0, 5.5, and 2.0 repetitions, and median ΔPCS = 0.0, −6.0, and −17.5 points for Minimal, Physical Function, and Psychological Responders, respectively; complete profiles across all outcomes are summarized in Table 5. The overall silhouette was 0.237 (C0 = 0.216; C1 = 0.171; C2 = 0.387), indicating moderate separation for C2 and weak‐to‐fair separation for C0 and C1. The gap statistic (*k* = 1–6), computed as the prespecified sensitivity check for the optimal number of clusters, favored *k* = 1 by Tibshirani's rule [33], so the 3‐cluster solution is presented descriptively and exploratorily for clinical interpretability rather than as an optimal solution.

## 5 Discussion

This study examined heterogeneity in response to a group‐based PNE‐PA program among older women with CLBP. Compared with a BM program, PNE‐PA yielded larger improvements in chair‐stand performance, daily step counts, and pain catastrophizing. Within the PNE‐PA group, three descriptive response patterns emerged: minimal responders, physical‐function responders, and psychological responders. Physical‐function responders showed greater gains in lower‐limb function and had lower baseline CS30 scores, whereas psychological responders showed larger reductions in catastrophizing and tended to present with lower baseline physical function and higher PCS scores.

Although no statistically significant differences in baseline PCS scores were found between clusters, analysis of score distributions provided additional insight. The median PCS score in the psychological responder group was 30.50, with more than half of the participants scoring at or above 30. A PCS score of 30 or higher is commonly considered indicative of clinically relevant catastrophizing [34, 35]. The lack of statistical significance may be attributable to limited sample size and intra‐cluster variance; nevertheless, the pattern of higher baseline PCS in psychological responders is clinically noteworthy and suggests that more pronounced catastrophizing may be linked to larger psychological change.

The observed psychological improvements align with previous studies demonstrating that PNE can effectively reduce maladaptive pain beliefs and catastrophizing in individuals with musculoskeletal pain [10, 11, 36]. The present findings extend this body of work by indicating similar benefits in older adults, who have been underrepresented in prior PNE research [12]. Dong et al. [37] also underscored the strong association between catastrophizing and psychological distress, such as anxiety and depression, among older adults with chronic pain. Taken together, these data support the rationale for interventions that explicitly target maladaptive beliefs and distress in this population, while recognizing that our cluster results remain exploratory.

In addition, the physical function responders demonstrated improved functional outcomes, suggesting that PNE‐PA can be associated with measurable gains in physical performance, particularly when functional deficits are present at baseline. This observation is consistent with the fear‐avoidance model, which proposes that pain‐related fear contributes to activity restriction and subsequent functional impairment [4, 5, 6, 38]. Camacho et al. [6] reported that fear‐avoidance beliefs strongly predict disability levels in older adults with CLBP. Although mediation was not formally tested, the pattern observed here is compatible with the idea that changes in pain‐related cognitions may facilitate greater engagement in physical activity and thereby contribute to functional improvement.

The pattern of psychological improvements without immediate physical gains observed in the psychological responder group may reflect a phased recovery process. According to the fear‐avoidance model, cognitive shifts such as reduced catastrophizing can decrease fear of movement and avoidance behavior, thereby setting the stage for later physical improvements [38, 39, 40]. The 12‐week intervention period may have been insufficient to capture such downstream changes in some participants, and longer‐term follow‐up could clarify whether early psychological change precedes subsequent functional gains.

The minimal responder group, characterized by relatively preserved baseline function and lower catastrophizing, may simply have had less scope for improvement in the outcomes measured. This does not imply that these participants would not benefit from other forms of support, but rather that the present program and outcome set captured limited change in this subgroup. Maladaptive psychological beliefs have been associated with lower physical activity levels, functional decline, frailty, and reduced quality of life in older adults with CLBP [2, 7, 37]. Early identification and targeted intervention for such beliefs may help prevent functional decline and preserve independence, although the current profiles are not yet ready to guide individual‐level treatment decisions.

There is growing evidence that central nervous system mechanisms play a role in mediating the effects of combined intervention [41]. Kregel et al. [41] documented structural and functional brain abnormalities in individuals with CLBP, suggesting central contributions to chronic pain. PNE may initiate cognitive reframing of pain‐related beliefs [10, 11], and when paired with gradual physical activity exposure, could support central adaptation through sensorimotor integration and behavioral activation [15, 16]. This dual pathway offers a plausible explanation for the early psychological improvements observed in some participants, but these mechanisms require direct investigation in future studies.

Daily step counts did not differentiate clusters and showed no cluster‐specific change, suggesting that simple ambulatory volume may not fully explain the observed response patterns. Step counts are a feasible and informative indicator of overall activity, yet they do not convey information on intensity, bout structure, or task‐specific exposure. These constraints, together with the quasi‐randomized design, modest cluster sizes, and only modest internal validity indices, indicate that our cluster‐level inferences should primarily be viewed as descriptive and hypothesis‐generating. Future studies should combine step counts with intensity‐based metrics and task‐specific activity monitoring and extend follow‐up to examine temporal ordering and potential mediators.

This study has several limitations. The quasi‐randomized design and small sample size, although sufficient for exploratory cluster analysis, limit causal inference and generalizability. The focus on older women restricts applicability to other groups, and the exclusion of regular analgesic users further narrows the target population. The short follow‐up period may have been inadequate to capture long‐term effects, particularly in psychological responders. In addition, clusters were defined using only two change variables (CS30 and PCS), which may have contributed to the emergence of domain‐based groupings and to sensitivity to regression toward the mean. Furthermore, covariate adjustment was restricted to age and BMI; inclusion of additional variables such as comorbidity count or sociodemographic factors was not feasible given the small sample size and the risk of model overfitting in this exploratory analysis. Future research should incorporate broader multidimensional assessments, prespecified primary outcomes, and larger samples to refine subgroup identification. Larger randomized studies with longer follow‐up are needed to confirm these findings and to test tailored combinations of PNE and progressive physical training protocols in older adults at risk for frailty. Finally, the interventions were implemented in different calendar periods, and potential seasonal or temporal influences on outcomes could not be fully controlled.

## 6 Conclusion

This study demonstrated that a group‐based PNE‐PA program was associated with improvements in lower‐limb function, physical activity levels, and pain catastrophizing in older women with CLBP, although individual responses varied. Cluster analysis identified three descriptive patterns of change: minimal responders, physical‐function responders, and psychological responders. Physical‐function improvements tended to occur in participants with lower baseline mobility, whereas larger psychological gains were observed in those with higher initial catastrophizing. These exploratory findings suggest that baseline physical and psychological status may be linked to the domain in which change occurs, but they are not yet sufficient to guide individual tailoring. Future adequately powered randomized studies are needed to confirm these patterns and to determine whether intervention content can be optimally matched to patient characteristics in community‐based programs for older adults with chronic pain.

## Author Contributions

**Teppei Abiko:** conceptualization, methodology, investigation, data curation, project administration, writing – original draft, funding acquisition. **Shin Murata:** conceptualization, methodology, writing – review and editing. **Hiroaki Iwase:** investigation, validation, writing – review and editing. **Koji Nonaka:** investigation, validation, writing – review and editing. **Kunihiko Anami:** investigation, validation, writing – review and editing. **Yuki Kikuchi:** investigation, validation, writing – review and editing. **Katsuyuki Madoba:** investigation, validation, writing – review and editing. **Kayoko Shiraiwa:** investigation, validation, writing – review and editing. **Guillermo de Castro Maqueda:** formal analysis, validation, writing – review and editing. **Wayne Hing:** validation, writing – review and editing, supervision.

## Conflicts of Interest

The authors declare no conflicts of interest.

## Transparency Statement

Teppei Abiko affirms that this manuscript is an honest, accurate, and transparent account of the study being reported; that no important aspects of the study have been omitted; and that any discrepancies from the study as planned have been explained.

## Data Availability

The data that support the findings of this study are available on request from the corresponding author. The data are not publicly available due to privacy or ethical restrictions. The data and analysis code will be available upon request.
